# Using Facebook to Reach People Who Experience Auditory Hallucinations

**DOI:** 10.2196/jmir.5420

**Published:** 2016-06-14

**Authors:** Benjamin Sage Crosier, Rachel Marie Brian, Dror Ben-Zeev

**Affiliations:** ^1^ Center for Technology and Behavioral Health Department of Biomedical Data Science Dartmouth College Lebanon, NH United States; ^2^ mHealth for Mental Health Program, Dartmouth Psychiatric Research Center Department of Psychiatry Dartmouth College Lebanon, NH United States

**Keywords:** hearing voices, auditory hallucinations, social media, Facebook, survey, advertisements

## Abstract

**Background:**

Auditory hallucinations (eg, hearing voices) are relatively common and underreported false sensory experiences that may produce distress and impairment. A large proportion of those who experience auditory hallucinations go unidentified and untreated. Traditional engagement methods oftentimes fall short in reaching the diverse population of people who experience auditory hallucinations.

**Objective:**

The objective of this proof-of-concept study was to examine the viability of leveraging Web-based social media as a method of engaging people who experience auditory hallucinations and to evaluate their attitudes toward using social media platforms as a resource for Web-based support and technology-based treatment.

**Methods:**

We used Facebook advertisements to recruit individuals who experience auditory hallucinations to complete an 18-item Web-based survey focused on issues related to auditory hallucinations and technology use in American adults. We systematically tested multiple elements of the advertisement and survey layout including image selection, survey pagination, question ordering, and advertising targeting strategy. Each element was evaluated sequentially and the most cost-effective strategy was implemented in the subsequent steps, eventually deriving an optimized approach. Three open-ended question responses were analyzed using conventional inductive content analysis. Coded responses were quantified into binary codes, and frequencies were then calculated.

**Results:**

Recruitment netted N=264 total sample over a 6-week period. Ninety-seven participants fully completed all measures at a total cost of $8.14 per participant across testing phases. Systematic adjustments to advertisement design, survey layout, and targeting strategies improved data quality and cost efficiency. People were willing to provide information on what triggered their auditory hallucinations along with strategies they use to cope, as well as provide suggestions to others who experience auditory hallucinations. Women, people who use mobile phones, and those experiencing more distress, were reportedly more open to using Facebook as a support and/or therapeutic tool in the future.

**Conclusions:**

Facebook advertisements can be used to recruit research participants who experience auditory hallucinations quickly and in a cost-effective manner. Most (58%) Web-based respondents are open to Facebook-based support and treatment and are willing to describe their subjective experiences with auditory hallucinations.

## Introduction

Seven percent of the population has experienced auditory hallucinations (eg, hearing voices) [1]. Many individuals experience auditory hallucinations in the context of a serious mental illness (SMI). Approximately 40% of people with SMI go undetected and untreated by traditional mental health care systems [2], a shortcoming exacerbated by environmental hurdles and poverty [3]. Many people with SMI report wanting to deal with their problems on their own rather than seek traditional treatment [2]. Novel outreach methods have the potential to reach individuals who experience auditory hallucinations and who are unwilling or unable to use traditional mental health services.

Traditional engagement methods for research through face-to-face contact at brick and mortar institutions, such as clinics or academic institutions, face numerous geographical and practical barriers (eg, stigmatization associated with going into a clinic, transportation). In contrast, social media offers an unparalleled opportunity to engage hard-to-reach populations, as most American adults (74%) use social networking sites [4], including those with a variety of health problems [5]. Facebook is the most successful Web-based social network to date, with nearly a billion and a half frequent users [6]. Other popular services like Instagram (400 million active users) and Snapchat (approaching 200 million active users), along with a plethora of smaller specialized networks, may offer additional opportunities for outreach [7]. These diverse user bases can be reached through advertising systems typically used for marketing through the promotion of posts, pages, apps, and Websites. Treatment information, Web-based surveys, and other recruitment materials can be distributed to vast audiences on modest budgets and within flexible timeframes in this advertisement ecosystem. Furthermore, advertising platforms have filters that allow for accurate targeting of personal characteristics, along with systems for real-time tracking of advertisement performance [8].

Facebook advertisements could be used as an effective recruitment tool for mental health studies, excelling in accessing hard-to-reach populations. Previous research has shown that most young people (94%) with SMI use social media [9], and most people with SMI use mobile technologies for communication and the Internet access [10]. Facebook has also been used to successfully recruit Veteran’s for mental health research [11]. Kosinski and colleagues offer a thorough general review of using Facebook as a research tool in any social science, highlighting this approach’s ability to rapidly collect data on millions of participants efficiently [12]. However, little is known about best approaches to reach those with SMI through the use of social media.

Previous research has demonstrated that overall survey length, topic, question length, sponsoring institution (eg, private industry, government, academia), all affect response rates of traditional surveys, but little work has been done to test these findings to Web-based surveys [13]. The success of using social media as an outreach or recruitment tool is partly determined by maximizing each step of the data collection process.

Advertisements need to be tested for relative effectiveness, honing in on materials that entice the most social media users to take action for the lowest cost to researchers. This approach is common practice in marketing. Advertisers commonly use “split tests” or “A/B testing” to hone their marketing strategies, on and off the Web [14]. Although general design principles guide advertisement design, the effectiveness of images is highly dependent on the nature of the audience and topic. Standard advertising practices suggest testing multiple advertisement layouts to determine efficiency, and the design of content is largely dependent on expert knowledge, in this case, auditory hallucinations.

Surveys need to be designed in a way that promotes conscientious and full participation at each step, discouraging incomplete responses and dropout. This research systematically tested these elements, arriving at an optimized advertisement and survey for people who experience auditory hallucinations. We detail the steps in this process and provide a cost analysis. Furthermore, we include insights gained from the survey itself regarding social media attitudes related to Web-based support and treatment. Specifically, we assess whether respondents would be willing to use social media to connect to peers experiencing similar problems or to clinicians to engage in services. We also present thematic frequencies from responses given by participants regarding what makes their hallucinations worse, ways in which they cope, and advice they would give to others who also experience auditory hallucinations.

## Methods

**Figure 1:**
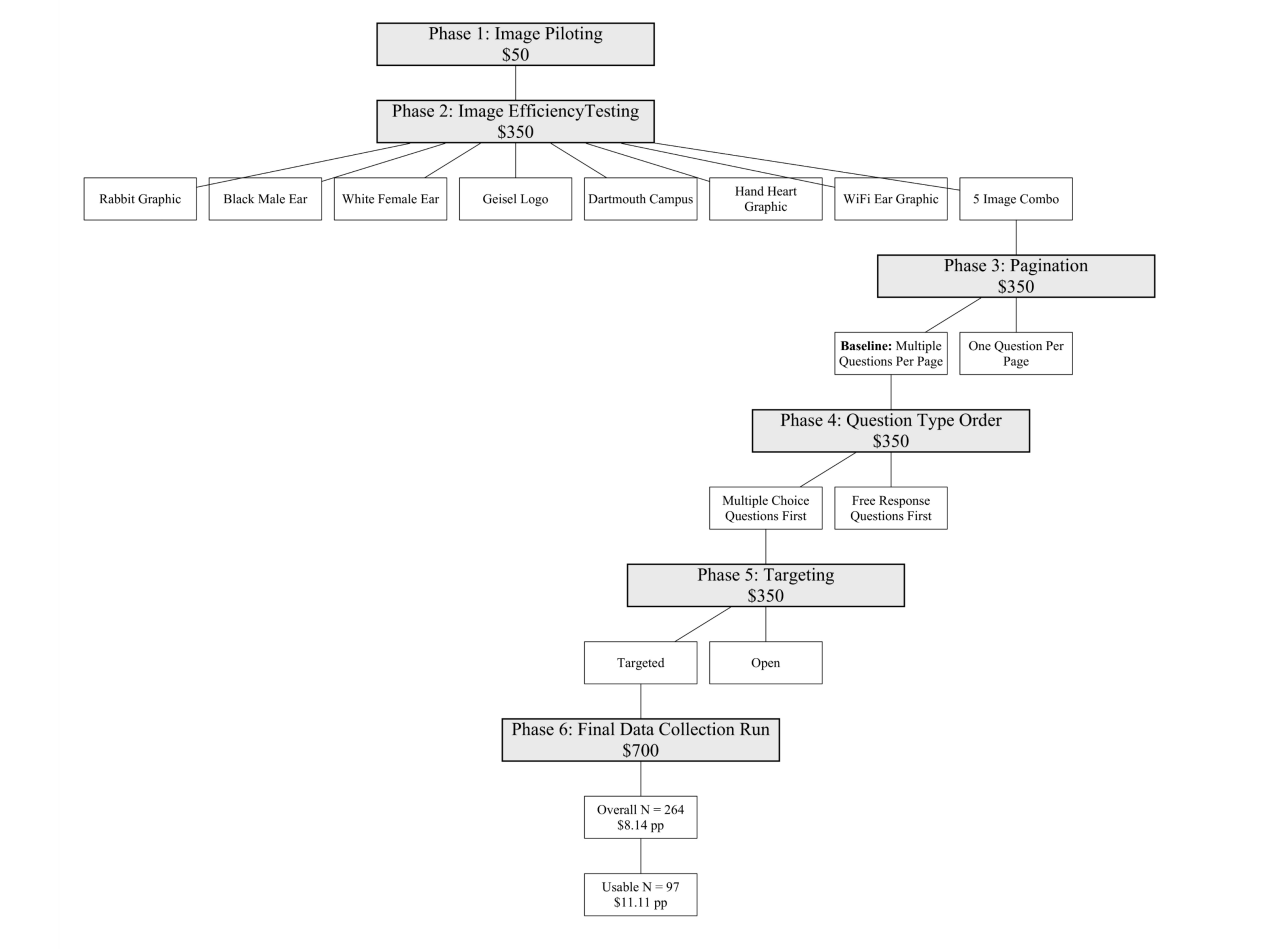
Engagement Optimization Phases.

The Committee for Protection of Human Subjects at Dartmouth College approved this study. Our team ran a series of Facebook advertisements in 6 phases (see Figure 1) to optimize response rate and data quality by distributing an 18-item survey (see Multimedia Appendix 1) between June 9 and July 17, 2015. We first piloted a set of candidate advertisements to ensure acceptability within Facebook’s advertising system. We then tested each of the images in 2 layout styles (single image vs a multiple image “carousel”) for relative performance. Next, we evaluated survey layout, maximizing data quality. We tested survey pagination, question type (multiple choice vs free response) ordering, and advertisement-targeting strategies. We concluded with a data collection run using optimized advertisement and survey designs to obtain a sufficient sample size.

**Figure 2:**
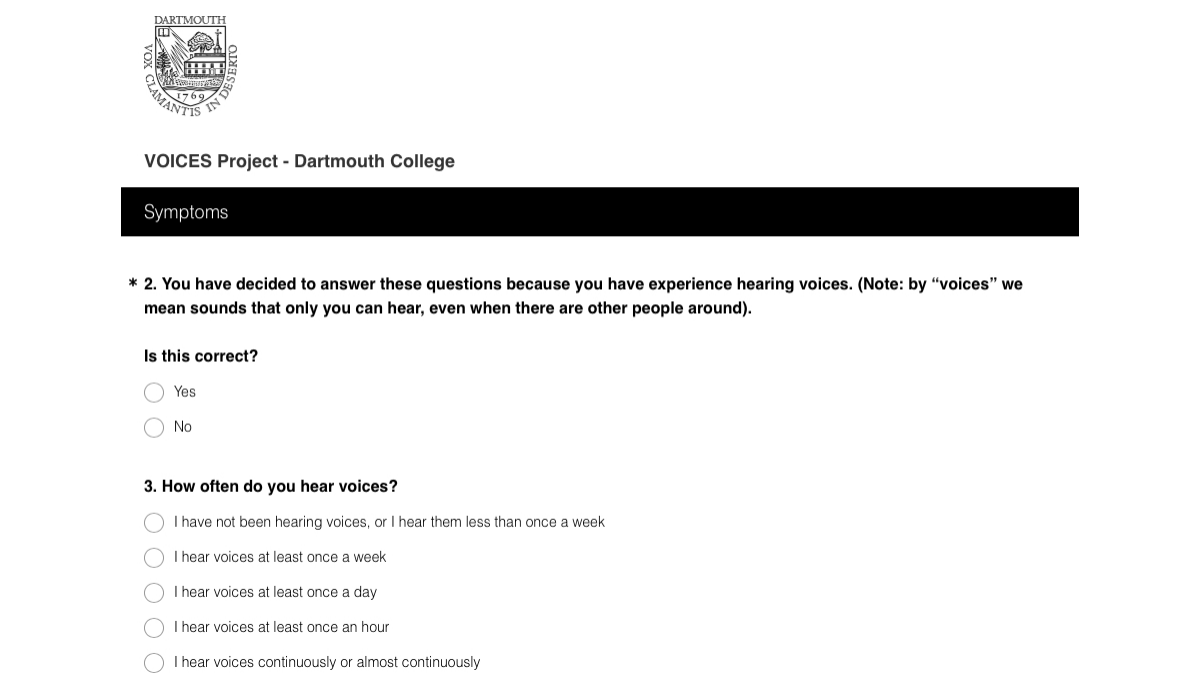
Screenshot of the survey platform.

Survey items captured demographics, symptom history, technology use, and attitudes, and solicited participant’s own experiences with auditory hallucinations and advice to help others (see Multimedia Appendix 1). To completely anonymize the survey, all automatic data collection features were turned off (eg, IP logging), and no identifying information was collected. Participants were eligible to participate if they were aged older than 18 years and confirmed that they experience auditory hallucinations, which was confirmed via survey questions (see Figure 2 for a screenshot of the survey platform hosted in Survey Monkey). Data from incomplete responses were logged by Survey Monkey, making it possible to determine click through rates. All advertisement distribution data, including cost, were monitored with Facebook’s advertising system.

Three open-ended questions were included in the Web-based survey, each having 3 fields for participants to provide responses. Questions included were *(1) What are 3 things that make your voices worse? (2) What are your top 3 methods for coping with voices? (3) What are 3 things people should keep in mind if they don’t want voices to control their lives?* Participant responses to the 3 open-ended questions were analyzed using conventional inductive content analysis [15]. Two research assistants (RW and GJ) with direct oversight from author (RB), independently read responses from each open-ended question to identify recurring regularities among entries to establish a preliminary list of codes [16]. Through group discussion and review, the induced codes were organized into categories to create the codebook [17,18]. Each research assistant then coded responses separately using this codebook. Once coding was complete, the 2 coders met to discuss their coded responses. Inter-rater reliability was calculated with 97.6% agreement. The rationale for why differently coded items were chosen was discussed until consensus was reached. Author RB then reviewed all coded content. When coding was finished, coded responses were *quantified* by converting responses to a binary format that was then used to calculate thematic frequencies [16]. Because there were 3 entries possible for each question, and to obtain a clear understanding of the unique responses given by each participant, similarly coded multiple responses given by a single participant were entered once into the database. For example, if an individual were to give the same response for each of the 3 entry fields for a question (eg, “ignore the voices,” “do not listen to the voices,” and “ignore”) these would be entered into the binary database for that participant and variable once. Thematic frequencies were then calculated from this database.

## Results

All data collection phases ($2150 total advertisement budget) collective garnered N=264 responses resulting in a final sample size N=97 participants that provided complete data. The cost per complete participant (N=97) in the final optimized stage was $8.14. The sample was 62% female with an average age of 28.91, standard deviation (SD)=14.50. The sample was composed of 57% white, 21% multiple race, 15% black, 4% American Indian or Alaskan Indian, and 3% Asian participants. A majority (82%) of the sample had at least a high school diploma or had obtained a General Educational Development (GED) certification. Participants were previously hospitalized for mental health problems M=1.97 (SD=4.57) times during their lifetime. Each phase of outreach optimization is sequentially described in the following sections. All quantitative data were analyzed with the R programming language. We include a series of tables to provide descriptives results on items not reported in main analyses. Tables 1 and 2 include self-reported information on diagnoses and symptom descriptions respectively. Table 3 covers treatment history, and Table 4 details technology use.

**Table 1:** Self-reported information on diagnoses.

Psychiatric diagnoses	Frequency	Percent
Schizophrenia	15	9.1
Schizoaffective	9	5.5
Bipolar	39	23.6
Depression	68	41.2
PTSD	29	17.6

**Table 2:** Frequency tables for mental health–related survey items.

Item	Response	Frequency	Percent
*“How often do you hear voices?”*						
	Not currently/less than once	39	24.4
	At least once a week	37	23.1
	At least once a day	39	24.4
	At least once an hour	10	6.3
	Almost continuously/continuously	35	21.9
*“How long have you been hearing voices?”*						
	<1 Month	4	2.5
	1-6 Months	12	7.5
	7-11 Months	15	9.4
	1-5 Years	38	23.9
	>5 Years	90	56.6
*“When they occur, how intense is your distress from the voices?”*						
	Not distressing	40	25.3
	Slightly distressing	46	29.1
	Moderately distressing	32	20.3
	Very distressing	28	17.7

**Table 3:** Treatment history.^a^

Response	Frequency	Percent
Medications	44	26.7
Group therapy	21	12.7
Individual therapy	39	23.6
Peer support	12	12.7
Mindfulness	32	19.4

^a^Participants also had the option of filling in a free-text “other” option. Two respondents listed “nothing” and the following items were each listed once: “control my surroundings,” “intensive inpatient and outpatient program,” “listening to them to see if I can help,” “marijuana,” ”meditations,” “music: the voices sing with me and are not tone deaf,” ”myself,” ”none,” ”not really, my other personalities dobt (*sic*) really let me,” ”psilocybin mushrooms.”

**Table 4:** Technology use–related items.

	Response	Frequency	Percent
Basic cell phone use			
	Do not have	53	42.7
	Every day	54	43.5
	4-6 days per week	5	3.2
	1 or less days per week	3	2.4
	Never use	10	8.1
Mobile phone use			
	Do not have	10	8.1
	Every day	104	83.9
	4-6 days per week	4	3.2
	1 or less days per week	2	1.6
	Never use	4	3.2
Tablet use			
	Do not have	63	52.5
	Every day	16	13.3
	4-6 days per week	3	2.5
	2-3 days per week	9	7.5
	1 or less days per week	13	10.8
	Never use	16	13.3
Wearable tech use			
	Do not have	78	47.3
	Every day	6	3.6
	4-6 days per week	2	1.2
	2-3 days per week	2	1.2
	1 or less days per week	2	1.2
	Never use	29	24.4
Email use			
	Do not have	8	4.8
	Every day	45	27.3
	4-6 days per week	15	9.1
	2-3 days per week	22	13.3
	1 or less days per week	23	13.9
	Never use	8	4.8
Social media use			
	Do not have	5	3.9
	Every day	101	79.5
	4-6 days per week	13	10.2
	2-3 days per week	5	3.9
	1 or less days per week	2	1.6
	Never use	1	0.8

### Phase 1

The initial phase of advertisement lasted 24-hours and ran on a budget of $50. This phase verified the acceptability of advertisement materials, should a particular image be deemed unacceptable by Facebook. Seven advertisement images and 2 advertisement layouts (single image vs a multiple image “carousel”) were tested, with 2 images not meeting Facebook’s standards because more than 20% of the image area was occupied by text. These images were altered to meet the 20% requirement. The baseline cost or the ratio of the number of times the advertisements were presented to the number of times it was clicked on, was then evaluated. See Table 5 for a breakdown of cost-per-click descriptives by phase. Table 6 provides data on cost per participant from the phases it was available, as well as a discussion of this availability in the Conclusion section.

### Phase 2

**Figure 3:**
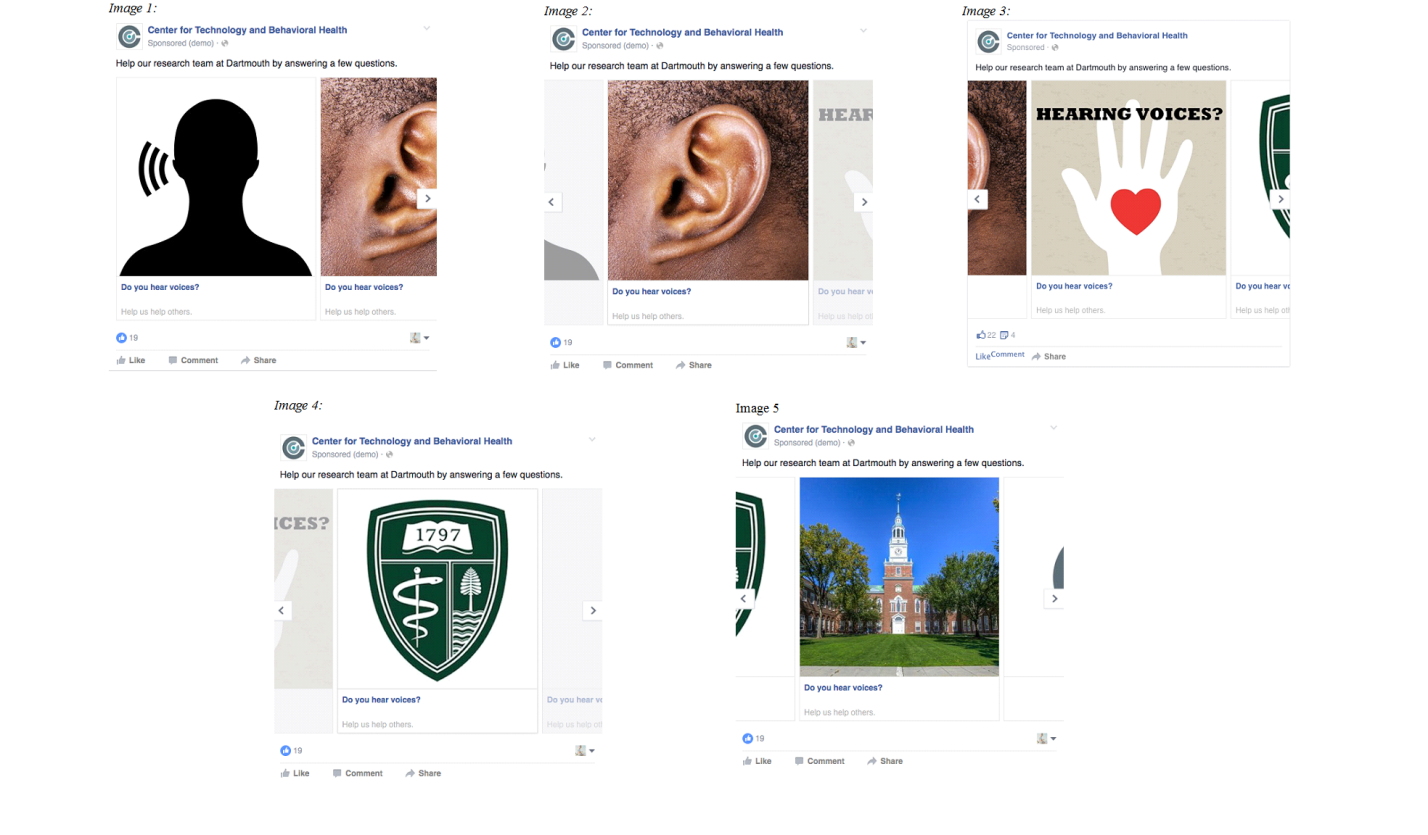
The final optimized advertisement. This advertisement was composed of 5 scrolling images that automatically rotated through a “carousel.” Five different perspectives of the advertisement are depicted to display each image. Facebook automatically optimizes image order for cost efficiency. Users can manually scroll through images by clicking right or left arrows.

The second phase ran over a 1-week period and had a budget of $350. This phase evaluated the relative cost-effectiveness of advertisement materials by simultaneously running each of the 7 single image advertisements and a version of the carousel-type advertisement (each of the 5 images in this advertisement were populated by the most cost-effective images in the image pilot; see Figure 3).

### Phase 3

This 1-week phase had a budget of $350. The optimized advertisement was used to test the influence of survey layout on response rate and data quality. This phase revealed no differences between grouping multiple questions per page versus having only one question on each page.

### Phase 4

A subsequent 1-week phase with a $350 budget revealed that data quality was increased by placing open-ended free response questions at the end of the survey rather than at the beginning.

### Phase 5

This 1-week phase used a budget of $350 to see if advertisement targeting could improve response rates. Facebook allows for the targeting of specific “interests.” These are calculated by Facebook with factors varying from demographics to purchase history, profile information, wall posts, news feed, and other communications (propriety method from Facebook). We selected a collection of promental health interests ranging from general terms (eg, schizophrenia) to Web-based support groups (see Textbox 1 for a list of each interest). This targeted approach outperformed an open approach with no filters.

**Table 5:** Cost per click and engagement by advertising phase.

Phase	Version	Reach	Clicks	CPC^a^
1: Image piloting	N/A	13,292	138	$0.36
2: Image efficiency	N/A	54,134	1,717	$0.20
3: Pagination	Multiple pages	1047	34,411	$0.17
	One page	1085	30,035	$0.17
4: Question type ordering	Multiple choice first	1277	35,644	$0.17
	Free response first	1175	32,494	$0.15
5: Targeting	Open	1379	31,095	$0.15
	Targeted	919	31,092	$0.19
6: Open data collection	N/A	112,122	3302	$0.20

^a^CPC: cost per click.

**Table 6:** Cost per participant (partial and complete responses together) by advertising phase.^a^

Phase	Version	N	Cost
1: Pagination	Multiple pages		$11.67
	One page		$9.72
2: Question type ordering	Multiple choice first		$9.72
	Free response first		$7.61
3: Open data collection	N/A		$6.03

^a^Cost data only available for phases that manipulated survey-related changes and not advertisement-related changes. This information was also available for the final open data collection phase (VI). Please see the conclusion section for a discussion of this limitation. (CPP=cost per participant).

**Facebook “Interests” used for advertisement targeting.**

Interests

Auditory hallucination

Bipolar disorder awareness

Delusional disorder

Depression and bipolar support alliance

Hallucination

Mental health

Paranoid schizophrenia

Psychiatry

Psychosis

Psychotherapy

Schizoaffective, depression, and bipolar support alliance

Schizoid personality disorder

Schizophrenia and smoking

Schizophrenia awareness

Schizophrenia research

Schizotypal personality disorder

Treatment of bipolar disorder

### Phase 6

A final 2-week data collection phase had a total budget of $700. This phase did not perform any optimization test but was instead used to collect a sufficient sample size of N=97 participants who provided fully completed responses.

Next, we used 2 multiple regression models that were specified a priori to examine the predictors of interest in using Facebook as a mobile mental health tool. We examined 2 outcome variables: interest in using Facebook to connect to similar others and interest in using Facebook to connect to clinicians. We included an identical set of predictors in each model that captured the level of distress from experiencing auditory hallucinations and technology use in each model, and controlled for demographics. Mobile phone use was predictive of an interest in using Facebook to connect with similar others (*b*=0.35, *t* (114)=2.18, *P*=.03). Females (*b*=−0.60, *t* (108)=−2.12, *P*=.04) and those who experienced more distress from hearing auditory hallucinations (*b*=0.30, *t* (135)=3.00, *P*=.003) expressed more interest in using Facebook to connect to clinicians.

### Qualitative Findings

Ninety-seven participants gave at least one response for one or more of the open-ended questions for qualitative analysis. Two participants were younger than 18 years and were removed from the dataset, along with a participant whose entries seemed disconnected from the questions asked (e.g., responding *“I am a wiccan”* as advice for others to help them with their auditory hallucinations, responding *"Marshall Law"* as something that makes their hallucinations worse). This gave a total of 94 eligible participants who gave at least one comprehendible open-ended response.

#### Auditory Hallucination Triggers

Out of the 90 people who responded, 209 unique entries were analyzed. Close to a third (31%) of responses related to some form of emotional distress (stress, anxiety, depression, or anger) as sources of symptom exacerbation. Social environments, such as being alone, situations involving other people, or conflict with others represented 23% of responses. Environmental factors (including noise, silence, light/darkness, and time of day) made up 15% of responses. Physical states, such as lack of sleep, excessive sleep, fever, and menstrual cycle accounted for 13% of unique responses. Seven percent of responses included alcohol, marijuana, or illicit substances. Mental states, such as overthinking/overfocusing or “zoning out” accounted for 5% of responses. Three percent thought that paying attention to auditory hallucinations and internalized stigmatization associated with auditory hallucinations (eg, *"Telling others about them,” “Thinking people have a reason to dislike me.”*) made their hallucinations worse. Three percent reported not knowing what made their hallucinations worse.

#### Ways Participants Cope With Auditory Hallucinations

One-hundred ninety eight codes were analyzed from 89 participants who gave responses. Most coping methods included strategies that could be used in the moment, including distraction techniques, ways of rationalizing hallucinations, and self-medication. Distraction techniques accounted for 58% of responses and included behavioral strategies such as listening to music, singing, socializing, relaxation techniques, sleep, exercise, and reading. Choosing to directly engage or disengage with the hallucinations, accepting them, rationalizing who/what they are, talking with or listening to hallucinations, or ignoring hallucinations accounted for 29% of responses. Five percent cope with some form of self-medication (alcohol, marijuana, or illicit substances), 3% cope with prescription medication, 2% use religion (prayer), and 3% did not know or reported that nothing makes their hallucinations better.

#### Advice Given to Help Others With Auditory Hallucinations

One-hundred seventy unique codes were analyzed from 80 participants who gave responses. Most responses (30%) dealt with understanding auditory hallucinations. This included the way one rationalizes who/what they are, the control one has over their auditory hallucinations, engaging with auditory hallucinations, acceptance of them or learning more about why they happen. Twenty-two percent of responses included finding distraction from the auditory hallucinations (ie, listening to music, relaxation techniques) or ignoring auditory hallucinations altogether. Thirteen percent included statements of encouragement such as, *“Remember that it could be worse”* and *“Stay strong.”* Nine percent of responses included suggestions to seek out mental health services or prescription medication. Another 9% listed that there was no help or that they did not know of any advice to give. Six percent of responses mentioned socializing, and another 6% contained religious statements (mostly about prayer). A small group advised the use of marijuana (2%) and 2% also listed other behavioral strategies (eat something, watch television, and so forth).

### Conclusion

We present results from a Web-based survey that used Facebook advertisements as a data collection strategy, suggesting that social media is a viable approach to reach people who experience auditory hallucinations. This scalable and efficient method can be used to tap a hard to reach population—a strength that is particularly relevant in reaching people who face economic or practical barriers accessing in-person services (eg, geographical) or are turned off by other outreach strategies that are plagued by the hazards of stigmatization.

Outside of recruitment and collection optimization, the exploratory survey provided insight surrounding personal experiences of those who experience auditory hallucinations and technology use. Regarding recruitment, we were able to obtain a larger proportion of minorities than is typically seen with traditional survey methods, without employing an oversampling strategy [19,20]. This is an especially important strength for research institutions that have limited access to diverse populations in their region. Inadequately representative sampling could be avoided by implementing an oversampling strategy within Facebook’s advertising system, partially blocking majority (eg, white, male, younger than 25 years) respondents during data collection.

Most participants reported that they were willing to use Facebook as a therapeutic tool. Most endorsed widespread use of technology, both in terms of devices and services. Participants also reported on a wide range of triggers to their auditory hallucinations, coping skills, and advice to others, providing qualitative insights that can be integrated into the design of technology-based treatment or support systems.

Our results were presented in 2 ways: the phased optimization of advertisement materials and survey design and the quantitative and qualitative results of the survey. First, we demonstrated that social media outreach strategies can be fine-tuned with systematic testing. Using multiple-image, “carousel”-style advertisements are suggested over single-image advertisements, and it is worthwhile to test a set of images for relative performance. Furthermore, advertisement targeting and restricting open-ended questions to the end of the survey had a positive effect, whereas survey pagination did not matter.

It is important to note that although correlated, that a low cost per click does not directly translate into high data quality. Engagement is necessary but insufficient to collect data from social media. A particular advertisement may generate a large number of clicks, but it not may net as many completed surveys as an advertisement or survey design that performs less well in terms of engagement, but encourages conscientious participation. It is also important to note that comparisons across advertising runs performed at different times are potentially inaccurate due to random fluctuations in advertising performance that are either random or caused by yet unexplored phenomena. Although general differences of large magnitude (eg, a cost per click of $0.10 vs $15.00) will probably hold at different times, smaller differences necessitate the need for comparisons across identical time frames. Comparisons across phases require large differences to be trustworthy, whereas comparisons within phases can be trusted even when the differences are much smaller.

A methodologic shortcoming did not allow us to obtain cost per participant within optimization phases where the factor being manipulated was on the side of Facebook’s advertising system, rather than the survey system, as seen in Table 6. Competing advertisements designs registered different tracking metrics within the advertising system, but a single survey was used to collect these data, which washed out the relationship between survey response rates and advertisement click rates. When surveys were compared, separate surveys were used, making these comparisons possible. Future research should consider using separate surveys (or “collectors” in Survey Monkey) to ensure that these data are captured.

Although the use of social media for recruitment offers tremendous new opportunities, the approach has noteworthy limitations. Differential use of social media is the foremost concern between those who have mental health problems and those that do not. More work needs to be done to explore the possible differences in social media use of this population as compared with those who are not experiencing symptoms. Although we know this group reports high social media use, their interaction patterns, use frequency, and posted content may be quite different. Future work should explore any potential differences.

Attention also needs to be paid to the fundamental motivation behind responding to a social media–based advertisement to participate in research. Whatever intrinsic or extrinsic motivational differences differentiate between those that click the advertisement and those that do not may be systematically related to social, psychological, emotional, behavioral, cultural, and demographic factors that impact the ultimate conclusions drawn from research performed with digital recruitment strategies. Regarding our study in particular, we only focused on the images in advertisements, and did nothing to modify the language used in the 25-character title and 90-character description allowed by Facebook. Future studies, complemented by empirically supported best practices from the fields of marketing and advertising, should test advertisement images and wording combinations in a multivariate fashion. Facebook’s advertising platform is well suited for such systematic testing with real-time tracking capabilities.

The rapid proliferation of social media has outpaced social and clinical scientists’ understanding of how these new resources can be harnessed to improve research, outreach, and services. Services like Facebook, Twitter, Snapchat, and Instagram offer an unprecedented way to reach populations of interest. These platforms automatically record an abundance of social data that can drive new research programs and inform the design of the next generation interventions. The development of best practices and new technology for massive outreach via social media and the collection of social media data itself should be a key focus of current research. This work suggests that engagement with social media advertising is a viable strategy for those that experience auditory hallucinations, hinting at the approach’s promise for those that experience other mental health symptoms that may be missed by traditional outreach systems.

## Appendix

Multimedia Appendix 1 Survey items.

## Abbreviations

SD: standard deviation
SMI: serious mental illness
