# Supplementary material for: Using Facebook to Reach People Who Experience Auditory Hallucinations
Source: J Med Internet Res. 2016 Jun 14;18(6):e160. doi: 10.2196/jmir.5420 (PMC4925933; doi:10.2196/jmir.5420)
Supplement: Multimedia Appendix 1 [file jmir_v18i6e160_app1.pdf]

You have decided to answer these questions because you have experience hearing voices. (Note: by "voices" we mean sounds that only you can hear, even when there are other people around). Is this correct?
-Yes
-No

How often do you hear voices?
-I have not been hearing voices, or I hear them less than once a week I hear voices at least once a week
-I hear voices at least once a day
-I hear voices at least once an hour
-I hear voices continuously or almost continuously

How long have you been hearing voices?
-Less than 1 month
-1 month - 6 months
-7 months - 11 months
-1 year – 5 years
-More than 5 years

When they occur, how intense is your distress from the voices?
-The voices are not distressing at all
-The voices are slightly distressing
-The voices are distressing to a moderate degree
-The voices are very distressing, although I could feel worse
-The voices are extremely distressing; I feel the worst I could possibly feel

If there were a Facebook resource that could help you connect with others who hear voices, would you use it?
-No
-Probably Not
-Maybe
-Probably
-Yes

If there were a Facebook resource that connected you with clinicians who could give you helpful tips for coping with voices, would you use it?
-No
-Probably Not
-Maybe
-Probably
-Yes

How often do you use the following?
-Basic cell phone

-Smartphone
-Tablet
-Computer (laptop, desktop)
-Wearable Tech (fitness tracker, fit bit, smart watch, etc.)
-Email
-Social Media (Facebook, Twitter)

Have you ever been diagnosed with any of these mental health conditions? (Please check all that apply)
-Schizophrenia
-Schizoaffective disorder
-Bipolar disorder
-Depression
-Posttraumatic stress disorder (PTSD)

Have you used any of the following treatments for voices? (Please check all that apply)
-Medications
-Group Therapy
-Individual Therapy
-Peer support
-Mindfulness
-Other (please describe)

How old are you?

What gender do you identify as?
-Female
-Male
-Other (please specify)

Which of the following best describes your race?
-White
-Black or African American
-Pacific Islander
-American Indian or Alaskan Native Asian
-More than one race

What is your highest level of education?
-No formal school
-Some elementary
-Finished 8th Grade
-Some H.S.
-HS Diploma /GED
-Some college/ technical
-Associates
-Bachelors

-Some graduate school
-Masters
-Doctorate

The next section will focus on helpful suggestions you may have for other people who hear voices. Please type in your answers in the spaces provided after each question.

How many psychiatric hospitalizations have you had in your lifetime?

What are 3 things that make your voices worse?
-1:
-2:
-3:

What are your top 3 methods for coping with voices?
-1:
-2:
-3:

What are 3 things people should keep in mind if they don't want voices to control their lives?
-1:
-2:
-3:
